# Case Report: Wiskott-Aldrich Syndrome Caused by Extremely Skewed X-Chromosome Inactivation in a Chinese Girl

**DOI:** 10.3389/fped.2021.691524

**Published:** 2021-07-08

**Authors:** Xuening Hou, Jie Sun, Chen Liu, Jihong Hao

**Affiliations:** ^1^Department of Clinical Laboratory, The Second Hospital of Hebei Medical University, Shijiazhuang, China; ^2^Department of Gynecological Ultrasound, The Second Hospital of Hebei Medical University, Shijiazhuang, China

**Keywords:** Wiskott-Aldrich syndrome, microthrombocytopenia, female carrier, heterozygous mutation, X-chromosome inactivation

## Abstract

Wiskott-Aldrich syndrome (WAS) is a rare X-linked immunodeficiency disorder caused by abnormal expression of Wiskott-Aldrich syndrome protein due to *WAS* gene mutation, which is generally characterized by microthrombocytopenia, eczema, recurrent infections, and high risk of autoimmune complications and hematological malignancies. Although affected males with WAS usually manifest severe symptoms, female carriers have no significant clinical manifestations. Here, we describe a Chinese girl diagnosed with WAS carrying a heterozygous missense mutation in exon 2 of the *WAS* gene. The patient presented with persistent thrombocytopenia with small platelets and decreased WAS protein detected by flow cytometry and western blot analysis. The methylation analysis of the *HUMARA* gene displayed an extremely skewed X-chromosome inactivation (SXCI) pattern, where the X-chromosomes bearing normal *WAS* gene were predominantly inactivated, leaving the mutant gene active. Hence, our results suggest that completely inactivating the unaffected paternal X-chromosomes may be the reason for such phenotype in this female patient. SXCI has important implications for genetic counseling of female carriers with a family history of WAS.

## Introduction

Wiskott-Aldrich syndrome (WAS, OMIM 301000) is a rare X-linked recessive immunodeficiency disorder characterized by thrombocytopenia with small platelets, eczema, and recurrent infections. The clinical phenotype has a wide spectrum, varying from mild thrombocytopenia to severe clinical manifestations, including life-threatening hemorrhages, immunodeficiency, atopy, autoimmunity, and malignancies (1). The *WAS* gene, whose mutations are associated with the clinical phenotype of WAS, is mapped to Xp11.23, and encodes for the Wiskott-Aldrich syndrome protein (WASp). The severity of WAS phenotype in patients correlates with specific mutations and the expression levels of WASp in the blood cells. Usually, affected males show symptoms at an early age, while females carrying the defective *WAS* gene are asymptomatic. Till date, fewer than 20 female cases have been reported with WAS worldwide (Supplemental Table 1). Most studies favor the hypothesis that the clinical phenotype of female carriers is attributed to skewed X-chromosome inactivation (XCI), where the mutated X-chromosomes are preferentially selected to be active (2–6).

Herein, we describe a 9-year-old Chinese girl with WAS who presented with congenital thrombocytopenia and hemorrhagic events. The patient had a heterozygous mutation of the *WAS* gene on the maternally derived X-chromosome, associated with the paternal X-chromosome completely inactive in the peripheral blood cells.

## Case Report

In 2019, a 9-year-old girl was admitted to the hospital because of severe colporrhagia for 3 days. She was initially admitted to the hospital due to thrombocytopenia at the age of 4 months and was diagnosed with immune thrombocytopenia (ITP). However, she failed to recover with hormone and human immunoglobulin injections, and her platelet count fluctuated between 20 × 10^9^/L and 30 × 10^9^/L. She then experienced frequent fever, followed by recurrent administration of anti-infective drugs due to upper respiratory tract infection. One and a half months ago, she was admitted to a local hospital for respiratory infection before being discharged with improved condition through anti-infection treatment.

Physical examination revealed petechiae on both eyelids and upper limbs, bulbous conjunctival hemorrhage and pharyngeal hyperemia, and tonsil I° swelling, although no eczema was noted. Peripheral blood cell count demonstrated 16.91 × 10^9^/L (3.5–9.5 × 10^9^/L) WBC, 139 g/L (120–140 g/L) hemoglobin, and 19 × 10^9^/L (125–350 × 10^9^/L) platelet with the low mean platelet volume (MPV) of 5.90 fL (7.4–11.0 fL). Small platelets were found in the peripheral blood smears.

Her serum IgG and IgM levels were lower than normal (3.78 and 0.14 g/L, respectively). Mycoplasma pneumoniae antibodies were positive, with the titer of 1:160, and without platelet-associated antibodies. Analysis of the lymphocyte subsets showed elevated total T cell count without any immunologic abnormalities. Bone marrow aspirate exhibited delayed maturation of megakaryocytes. Combined with her medical history and ineffective hormonal treatment, she was suspected of having inherited platelet disorders.

**Figure 1:**
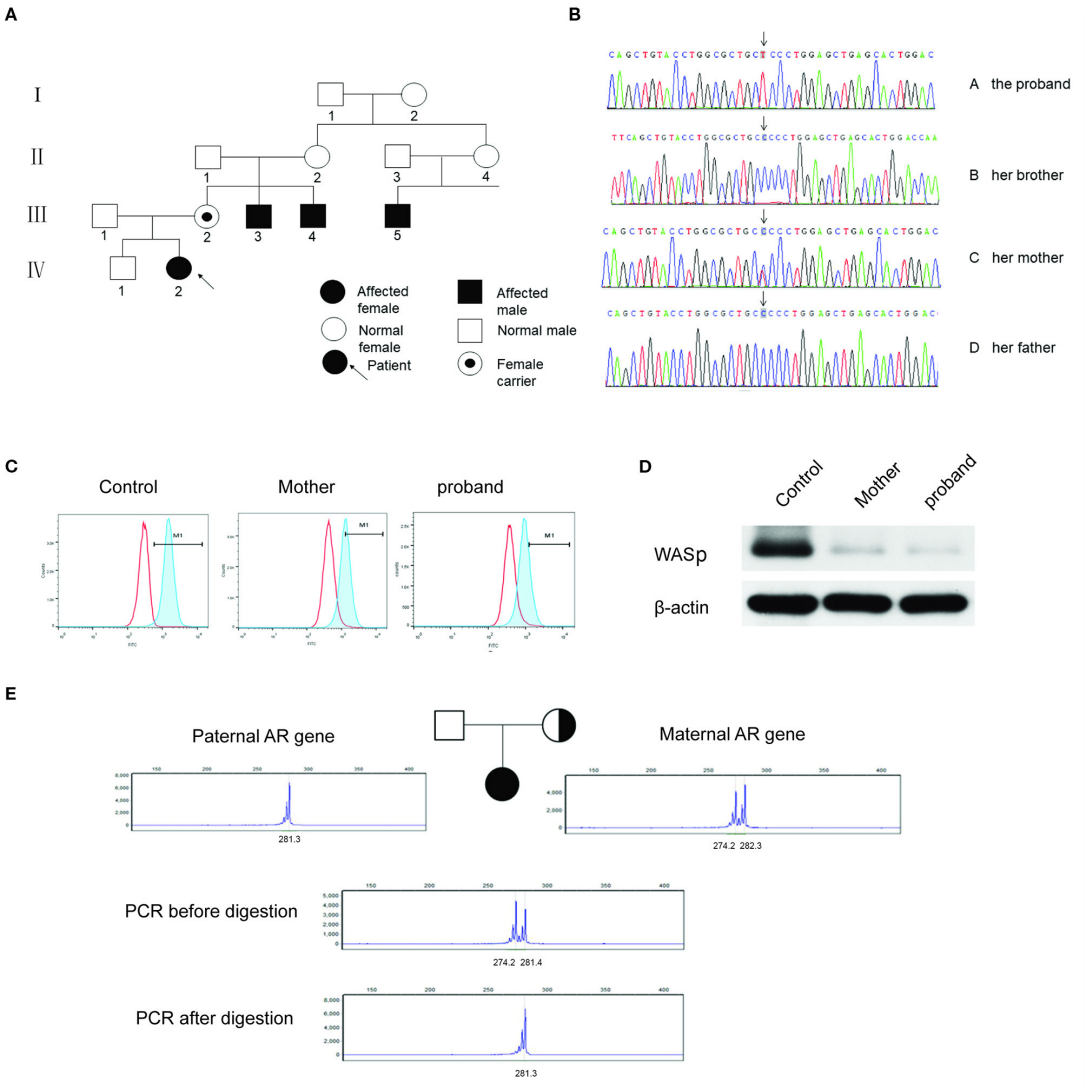
**(A)** The pedigree of the patient's family. III.2: female carrier; III.3, III.4, and III.5: affected male; IV.2: female proband. **(B)** Sanger sequencing of the *WAS* gene of c.173C>T. The proband and her mother were heterozygous of the mutated gene, and her older brother and her father were normal. **(C)** WASp expression levels in the PBMCs by flow cytometry assay. The proportion of M1 area was 98.5% of normal control, 50.6% of her mother and 20.6% of the proband, respectively. **(D)** WASp expression levels were analyzed by western blotting. The gray mean value of the proband's mother is 10.8% of the control level and the gray mean value of proband is 5.4% of the level. **(E)** X-chromosome inactivation analysis of the patient and her parents. The experiments were performed in triplicate.

Upon investigating her family history, her father and elder brother showed no symptoms (Figure 1A), while her mother showed decreased IgM and IgA levels, with normal PLT. The detailed results are shown in Table 1. Her three maternal uncles presented thrombocytopenia for many years.

**Table 1:** Immunologic and hematologic characteristic of the patient and her mother.

**Variable**	**Patient**	**The patient's mother**	**Normal range**
**Platelets**			
Count (× 10^9^/L)	19	270	125–350
Mean Volume (fL)	5.9	9.1	7.4–11.0
**Serum immunoglobins (g/L)**			
IgG	3.78	11.97	7.5–15.0
IgM	0.14	0.52	1.0–5.0
IgA	1.74	0.34	0.46–3.0
**Lymphocyte phenotype (%)**			
Total T cells	77.9	66.9	20–50
CD3+CD4+	48.7	38.5	33–58
CD3+CD8+	26.9	21.7	20–39
CD3+CD4+/CD3+CD8+	1.81	1.77	0.7–2.5
CD19+/CD20+	9.9	7.6	5–18
CD3-CD56+	11.4	23.7	10–20

**Figure 2:**
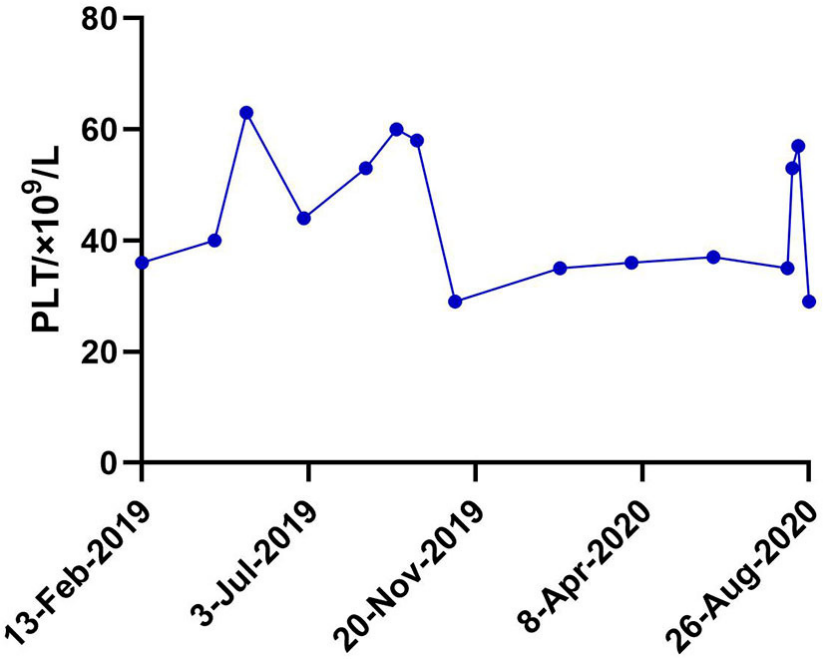
Platelet fluctuations of the patient within one and a half years (13-Feb-2019–26-Aug-2020) after admission.

Sanger sequencing revealed a heterozygous missense mutation in exon 2 of the *WAS* gene, leading to a change from proline to leucine at position 58 in the patient and her mother (Figure 1B). No mutations were detected in her father or elder brother. Meanwhile, flow cytometry showed the level of WASp from the proband and in the cells from her mother at 20.6 and 50.4%, respectively (Figure 1C). The ratio of WASp was reduced by ~5-fold in mononuclear cells from the proband compared to the normal control (98.5%). Western blot analysis revealed significantly decreased WASp levels in the patient and her mother compared to normal control (Figure 1D). Based on the above findings, the girl was confirmed to have WAS. Her platelet count fluctuated between 30× 10^9^/L and 60× 10^9^/L within one and a half years after admission (Figure 2). At present, the patient is receiving symptomatic treatment and awaiting hematopoietic stem cell transplantation.

Moreover, XCI analysis was performed using the human androgen receptor assay (HUMARA). Briefly, genomic DNA was extracted from peripheral blood leukocytes and used to amplify the highly polymorphic CAG repeat in the first exon of the *HUMARA* gene on Xq11-q12 (4). The degree of X-inactivation was calculated from the peak height. The results showed that the proband had only one paternal allele amplified after *HpaII* digestion (Figure 1E). In contrast, her mother showed two bands in the presence of *HpaII*; however, the rate of random inactivation indicated a mildly skewed pattern of XCI in mature blood cells (Figure 1E). The rate of XCI was 100 and 62.7% for the patient and her mother, respectively (Table 2).

**Table 2:** The results of X-chromosome inactivation of the patient and her mother.

	**PCR fragment location (-)**	**PCR fragment location (+)**	**Peak 1** **height (+)**	**Peak 2 height (+)**	**Peak 1** **height (-)**	**Peak 2 height (-)**	**Rate of random inactivation**	**The proportional relation of random inactivation**
IV2	n1 274.2	/	0	1,301	580	770	100%	100:0
	n2 281.4	281.3						
III2	n1 274.2	274.1	969	824	1,173	1,686	62.8%	63:37
	n2 282.3	282.1						
III1	n1 281.3							

*-, before digestion by HpaII; +, after digestion by HpaII; n1/n2, the locations of two alleles; IV2 means female proband, and III2 means the proband's mother, a female carrier and III1 means the proband's father; the length of the corresponding allele peaks in the proband represents the paternal allele (281nt) and maternal allele (274nt). The degree of random inactivation: 50:50~59:41: complete random X-chromosome inactivation; 65:35~79:21: mild skewed X-chromosome inactivation; >80:20: serious skewed X-chromosome inactivation*.

## Discussion

Herein, we present a Chinese girl with a congenital thrombocytopenia, hemorrhagic tendency, and recurrent infections with c.173C>T, a missense mutation of the *WAS* gene. Although missense mutations are most common in patients with WAS, this mutation is so rare that only three cases have been reported in the USA, China, and Brazil (7–9). Unlike the three male cases, our patient was a girl with this mutation. The c.173C>T mutation reported here is in the second exon that falls in the WH1 domain, which binds to the site of WASp interacting protein (WIP), crucially influencing the stability of WASp. Moreover, this domain plays a critical role in a series of events, including intracellular signal transduction, protein folding, and transport (10).

The *WAS* gene located on the X-chromosome contributed to WAS, which is transmitted in compliance with an X-linked recessive inheritance feature. X-linked female carriers show no clinical manifestations due to the pattern of XCI, which may ameliorate the destructive effects of the X-linked mutations (11). Nevertheless, XCI maintenance mechanisms are remarkably diverse, which closely affects X-linked gene dosage and sex differences in X-linked diseases (12). Therefore, the pattern of XCI may aggravate the clinical manifestations of the disease. Our present study proposed that skewed XCI led to the clinical phenotype of this female patient with WAS. In some cases, this is possibly due to genetic defects in the X-inactivation transcriptional process, contributing to the unusually skewed XCI pattern (4). The imbalanced expression of the parental X-chromosomes seems to be common in the general population; however, the skewed XCI pattern increased the risk of recessive X-linked disorders in females (13).

The results of XCI analysis showed two peaks at different positions in the proband and her mother before digestion with *HpaII*, indicating that the alleles were derived from both paternal and maternal chromosomes. However, the proband had only one allele left after enzymatic digestion, demonstrating that the proband had preferential inactivation of the paternally derived wild-type allele. The selection of XCI was independent of the mutation originating from either parent. While the spontaneous heterozygous mutation of the *WAS* gene was on the paternally derived X-chromosome, the extremely skewed XCI preferentially selected the maternally derived wild-type X-chromosome to be inactivated (5). From a comprehensive perspective, the genetic basis of the clinical presentation of WAS in our proband is the fortuitous combined occurrence of a maternally inherited mutation and the extreme skewing of inactivation against the unaffected paternal chromosome.

The proportion of inactivation was 100% in the patient and 62.7% in her mother (Table 2), manifesting the overwhelmingly skewed XCI of the patient and slight inactivation of her mother. These results were consistent with the expression levels of WASp. At the cytogenetic level, it also explained the differences in clinical signs between the mother and daughter, who were both carriers of heterozygous mutation.

Based on WASp-deficient mice, the skewed inactivation of WAS carriers was attributed to homing deficits in fetal hematopoietic stem cells (14). In addition, the selected inactivation can be present at a later stage, even in mature populations of hematopoietic differentiation (15). Thus, the predominant selection against mutated alleles as well as skewed inactivation can occur throughout the progress of hematopoietic development, increasing the risk of WAS in females. Diverse patterns of XCI exist in female carriers, such as random, slightly skewed, or extremely skewed patterns (3, 16). As a heterozygous carrier, the patient's mother exhibited slightly skewed inactivation in our study. This illustrates that the XCI pattern in humans shows diversity between individuals without stochastic mechanisms. In addition, a previous study indicated that the blood cell population bearing the mutated *WAS* message was greater in patients with WAS than in their mothers (3). Hence, the degree of imbalanced XCI in WAS carriers may vary with age. A WAS carrier has been reported to present random XCI in granulocytes and non-random inactivation in T lymphocytes (15). In contrast, a female with X-linked thrombocytopenia was also reported with random inactivation in lymphocytes along with skewed XCI in buccal mucosal cells (17). Therefore, the XCI pattern also varies at the cellular level and across different cell types.

In summary, the cumulative clinical results and molecular assays illustrated that a female carrier with a heterozygous *WAS* mutation could express an X-linked WAS disease. To the best of our knowledge, the case reported here is the first Chinese female patient presenting WAS clinical symptoms with an extremely skewed XCI pattern, in which the mutated allele was preferentially active. XCI analysis is a relatively effective and rapid technique to analyze the pattern of XCI in female carriers, who are suspected of having an X-linked recessive disorder. Furthermore, this case reminds us of the fact that female carriers with a family history of WAS may also develop certain clinical symptoms.

## Data Availability Statement

The original contributions presented in the study are included in the article/Supplementary Material, further inquiries can be directed to the corresponding author/s.

## Ethics Statement

The studies involving human participants were reviewed and approved by the Research Ethics Committee of the Second Hospital of Hebei Medical University. Written informed consent to participate in this study was provided by the participants' legal guardian/next of kin. Written informed consent was obtained from the individual(s), and minor(s)' legal guardian/next of kin, for the publication of any potentially identifiable images or data included in this article.

## Author Contributions

All the authors have accepted responsibility for the entire content of this submitted manuscript and approved submission. XH drafted the manuscript. JS and CL contributed to the design, acquisition, and analysis of data. JH final approval of the version to be published and offered professional guidance.

## Conflict of Interest

The authors declare that the research was conducted in the absence of any commercial or financial relationships that could be construed as a potential conflict of interest.
