# Supplementary material for: Case Report: Wiskott-Aldrich Syndrome Caused by Extremely Skewed X-Chromosome Inactivation in a Chinese Girl
Source: Front Pediatr. 2021 Jul 8;9:691524. doi: 10.3389/fped.2021.691524 (PMC8295588; doi:10.3389/fped.2021.691524)
Supplement: Supplementary file 1 [file Data_Sheet_1.zip › Data Sheet 1/Supplementary Material Presentation/Figure 2.docx]

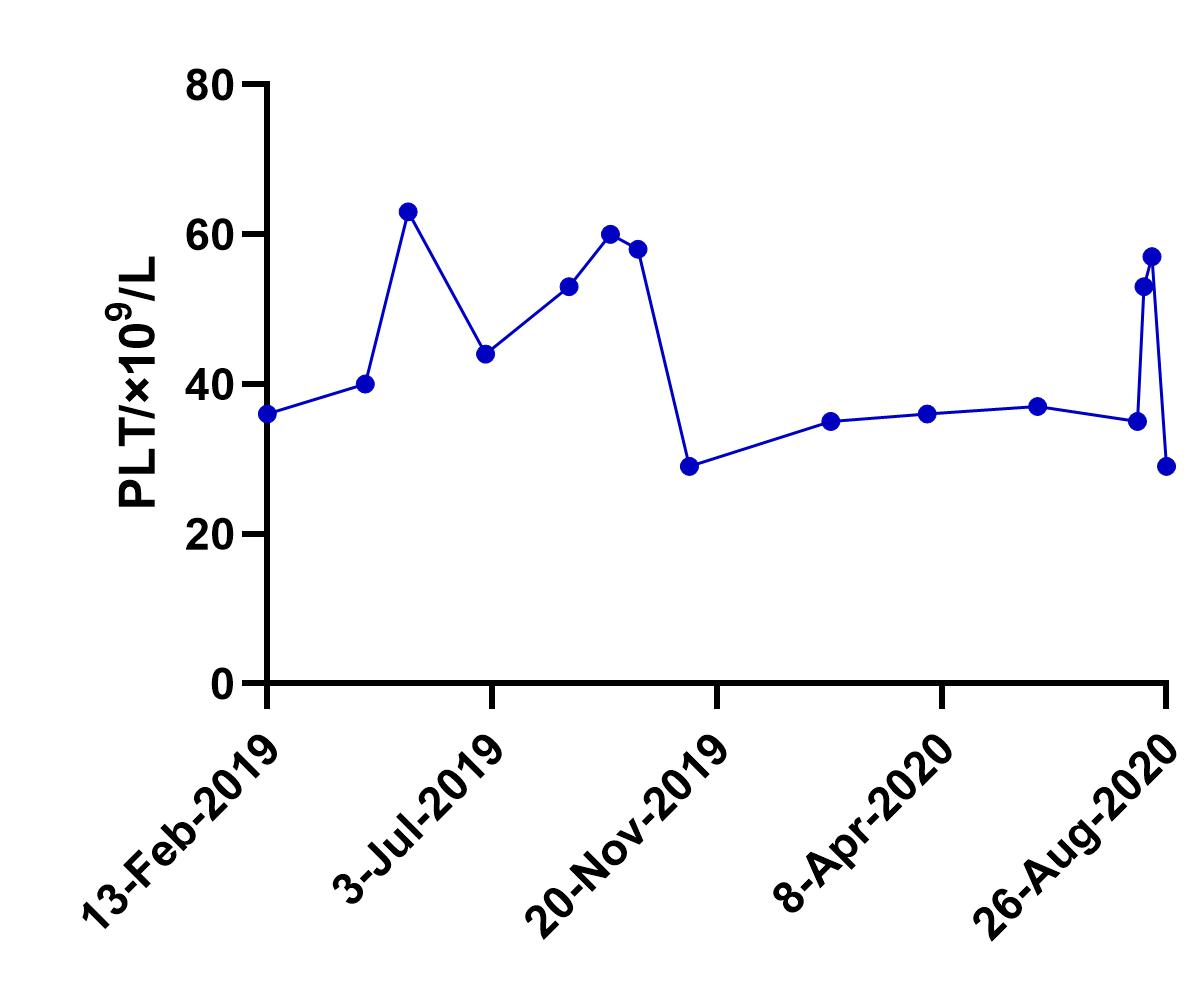


**FIGURE 2|** Platelet fluctuations of the patient within one and a half years (13-Feb-2019 to 26-Aug-2020) after admission.
