# Supplementary material for: Case Report: Wiskott-Aldrich Syndrome Caused by Extremely Skewed X-Chromosome Inactivation in a Chinese Girl
Source: Front Pediatr. 2021 Jul 8;9:691524. doi: 10.3389/fped.2021.691524 (PMC8295588; doi:10.3389/fped.2021.691524)
Supplement: Supplementary file 1 [file Data_Sheet_1.zip › Data Sheet 1/Supplementary Material Presentation/Supplementary Table 1.docx]

**SUPPLEMENTARY TABLE 1|** XLT/WAS in females reported with X-chromosome inactivation.

| **Reference** | **subject** | **Family history** | ***WAS* mutation** | **WAS protein level** | **Clinical features** | **X-chromosome inactivation analysis** | **DOI** |
| --- | --- | --- | --- | --- | --- | --- | --- |
| Greer et al (1989) | 25 females from 10 kindreds | Yes | Unknown | Unknown | Unknown | Nonrandom X-chromosome inactivation in obligate carriers | 10.1016/0888-7543(89)90315-7 |
| Notarangelo et al (1991) | a female from a pedigree | Yes | Linkage analysis (DXS255) | Unknown | Mild thrombocytopenia with small and normal-sized platelets | Nonrandom X- inactivation in T-lymphocytes; random X-inactivation in granulocytes | 10.1007/bf00206081 |
| Conley et al (1992) | an 8-year-old girl | No | Unknown | Unknown | Thrombocytopenia, eczema, recurrent infections | Random X-chromosome inactivation | 10.1182/blood.V80.5.1264.1264. |
| Russell et al (1995) | a 15-month-old girl | No | Unknown | Unknown | Thrombocytopenia, eczema, recurrent infections | Random X-chromosome inactivation | 10.1111/j.1365-2141.1995.tb03403.x |
| de Saint Basile et al (1996) | two females | Yes | p.V75M | Unknown | Thrombocytopenia | Random X-chromosome inactivation | 10.1016/s0022-3476(96)70190-7 |
| Parolini et al (1998) | a two-month-old girl | No | c.431 G>A; p.E133K | Unknown | Thrombocytopenia, eczema, immunodeficiency | Skewed X-inactivation with the mutated X-chromosome preferentially active | 10.1056/nejm199801293380504 |
| Ariga et al (1999) | 15 females from 10 families | Yes | 10 carriers with various *WAS* mutation | Unknown | Small-sized platelets | Extremely skewed X-inactivation for seven females; skewed X-inactivation for two carriers | 10.1046/j.1365-2141.1999.01248.x |
| Inoue et al (2002) | a 6-year-old girl | Yes | IVS 6+5G>A | one third that of the normal control | Thrombocytopenia, Petechiae | Random X- inactivation in peripheral blood cells; skewed X-inactivation in buccal mucosal cells | 10.1046/j.1365-2141.2002.03740.x |
| Lutskiy et al (2002) | a 14-month-old girl | Yes | IVS 6-1G>A | 60% of normal level | Thrombocytopenia, petechiae | Random X-chromosome inactivation | 10.1182/blood-2002-02-0388 |
| Andreu et al (2003) | a 19-year-old girl | Yes | c.291 G>A; p.R86H | Unknown | Mild thrombocytopenia with small and normal-sized platelets | Skewed X-inactivation with the normal X-chromosome preferentially inactive | 10.1016/s1079-9796(03)00168-2 |
| Boonyawat et al (2013) | a 9-month-old girl | No | c.397 G>A; p.E133K | no expression | Thrombocytopenia, eczema, reduced weight | Extremely skewed X-chromosome inactivation | 10.1007/s10875-013-9927-9 |
| Daza-Cajigal et al (2013) | 6 females from a family | Yes | p.V332A | Normal | Moderate-severe thrombocytopenia, petechiae | Completely skewed inactivation of the paternal X-chromosome with wild-type gene | 10.1016/j.bcmd.2013.04.004 |
| Takimoto et al (2015) | a 6-month-old girl | No | c.1276_1285 del_GCCCCTG GTG; p.Ala426GlyfsX15 | Defective expression | Thrombocytopenia, eczema, intestinal bleeding | Nonrandom X-inactivation with the paternally X-chromosome carring wild-type gene preferentially inactive | 10.1159/000370059 |
| present study | a 9-year-old girl | Yes | c.173 C>T; p.P58L | Defective expression | Severe thrombocytopenia with small platelets, petechiae, vagina bleeding, recurrent infections | Extremely skewed X-chromosome inactivation with mutated X-chromosome preferentially active | _ |
