# Supplementary material for: Case Report: Wiskott-Aldrich Syndrome Caused by Extremely Skewed X-Chromosome Inactivation in a Chinese Girl
Source: Front Pediatr. 2021 Jul 8;9:691524. doi: 10.3389/fped.2021.691524 (PMC8295588; doi:10.3389/fped.2021.691524)
Supplement: Supplementary file 1 [file Data_Sheet_1.zip › Data Sheet 1/Supplementary Material Presentation/Therapeutic intervention and method description.docx]

1. **Therapeutic intervention**

Due to limited clinical data, we can only obtain the content of therapeutic interventions when the patient was suspected of hereditary thrombocytopenia. At that time, our patient was treated with traditional Chinese medicine decoction, Dihuang Zhixue Capsule, iron succinate tablets, Drospirenone and Ethinylestradiol tablets and azithromycin dry suspension orally. Mannan peptide was applied for improving her immunity. Platelet fluctuation was from 27×10^9^/L to 36×10^9^/L and the condition was stable. Hence, the patient was discharged from hospital with medication.

1. **Patient perspective**

The patient and her family were informed of the disease and agreed to the therapeutic schedule.

1. **Complete methods files**

**3.1. Flow cytometry**

Heparinized blood samples were collected and processed from the patient, her mother, and normal control. Peripheral blood mononuclear cells (PBMCs) were isolated by standard Ficoll-Hypaque gradient centrifugation methods. Intracellular staining with anti-WASp monoclonal antibody (mAb) was performed as previously described. Cells were incubated with 0.25mg/ml purified mouse anti-human WASp mAb (BD pharmingen, Franklin Lakes, NJ) or 0.5mg/ml isotype-matched control mouse IgG2a mAb (BioLegend, San Diego, CA) and reacted with 1:100 diluted fluorescein isothiocyanate (FITC)-conjugated goat anti-mouse IgG2a (Affinity Biosciences, Jiangsu, China). The samples were analyzed on BD FACSCanto Ⅱ (Becton Dickinson, Franklin, Lakes, NJ), using FlowJo V10 software (Becton Dickinson).

**3.2. Western blotting**

PBMCs were isolated by standard Ficoll-Hypaque gradient centrifugation methods and washed twice by PBS and lysed with RIPA buffer. The total protein was extracted from cell lysates, 20ug of which was separated and electrophoresis by 10% SDS-PAGE, then transferred to PVDF membranes. Blocked with 5% nonfat milk in TBST, the membranes were incubated with anti-WASp mAb (1:500; BD pharmingen, Franklin Lakes, NJ) as the primary antibodies and anti-β-actin antibodies (1:3000; Servicebio, Wuhan, China) as a control for protein loading at 4℃ for 12h. Washed by TBST three times, the membranes were incubated with goat anti-mouse IgG-HRP (1:500; Affinity Biosciences, Jiangsu, China) at 25℃ for 1h. Then the electrochemical luminescence was used to develop and fix the images. The optical density of the target band was analyzed by Alpha software.

**3.3. Sequencing analysis**

Genomic DNA was extracted from blood samples of the patient family members by using the DNA Isolation Kit for Mammalian Blood (Tiangen Biotech, Beijing, China) and the samples were amplified by polymerase chain reaction and then sequenced by the next-generation sequencing (NGS) screening the inherited platelet disorders (IPD) and immunodeficiency diseases. The abnormal sites of *WAS* gene were sequenced by sanger analysis. The results were analyzed using Mygenostics software and mutations were identified by comparing with the genomic gene of *WAS* (NG-007877.1) in GeneBank's human genome database.

**3.4. X-chromosome inactivation analysis**

The analysis of XCI was performed by human androgen receptor assay (HUMARA). Briefly, genomic DNA was extracted from peripheral blood leukocytes and used to amplify the highly polymorphic CAG repeat in the first exon of the *HUMARA* gene on Xq11-q12. To clarify the XCI patterns, PCR products were obtained to make fragment analysis with ABI 3130 sequencer and then analyzed by GeneMapper software. The X-inactivation degree was calculated from peak height. The formula of random X-inactivation rate is as follows:

The rate=(d1/u1)/(d1/u1+d2/u2) ×100%.

d1 and d2: the height of peak1 and peak2 after digestion with *HpaⅡ*; u1 and u2: the height of peak1 and peak2 before digestion with *HpaⅡ*. The PCR products were subjected to capillary electrophoresis and modified polyacrylamide gel electrophoresis.
